# Investigation of the Localized Corrosion and Passive Behavior of Type 304 Stainless Steels with 0.2–1.8 wt % B

**DOI:** 10.3390/ma11112097

**Published:** 2018-10-25

**Authors:** Heon-Young Ha, Jae Hoon Jang, Tae-Ho Lee, Chihyoung Won, Chang-Hoon Lee, Joonoh Moon, Chang-Geun Lee

**Affiliations:** 1 Ferrous Alloy Department, Korea Institute of Materials Science, Changwon 51508, Korea; jhjang@kims.re.kr (J.H.J.); lth@kims.re.kr (T.-H.L.); kta2017@kims.re.kr (C.W.); lee1626@kims.re.kr (C.-H.L.); mjo99@kims.re.kr (J.M.); lcg0827@kims.re.kr (C.-G.L.); 2 Department of Metallurgical Engineering, Pusan National University, Busan 46241, Korea

**Keywords:** borated stainless steels, pitting corrosion, passive film

## Abstract

The pitting corrosion resistance and passive behavior of type 304 borated stainless steels (Fe_balance_–18Cr–12Ni–1.5Mn–(0.19, 0.78, and 1.76 wt %)B) manufactured through conventional ingot metallurgy were investigated. The alloys were composed of an austenitic matrix and Cr_2_B phase, and the volume fraction of Cr_2_B increased from 1.68 to 22.66 vol % as the B content increased from 0.19 to 1.76 wt %. Potentiodynamic polarization tests measured in aqueous NaCl solutions revealed that the pitting corrosion resistance was reduced as the B content increased and the pits were initiated at the matrix adjacent to the Cr_2_B phase. It was found that the reduced resistance to pitting corrosion by B addition was due to the formation of more defective and thinner passive film and increased pit initiation sites in the matrix.

## 1. Introduction

Boron (B)-containing austenitic stainless steels known as borated stainless steels (BSSs) have been widely used in the nuclear industry primarily due to their excellent thermal neutron attenuation capability. The BSSs are used in storage racks and transportation casks for the storage of spent nuclear fuel from power reactors [1,2,3,4,5,6,7,8,9,10,11]. Because the neutron absorption ability directly relies on the B content in the materials [3,6,8,10], the BSS should contain as much B as possible to be safely used as wet storage rack materials for spent nuclear fuel. In addition, desirable strength, formability, and corrosion resistance in a wet storage environment are also required. Eight types of BSSs based on AISI S30400 stainless steel, which meet the requirements of the mechanical properties, are specified in ASTM A 887–89 (ASTM International, West Conshohocken, PA, USA, 2004a) [12] from 304B with 0.20–0.29 wt % B to 304B7 with 1.75–2.25 wt % B [2,3,7,8,9,10,11].

A small addition of B of less than approximately 50 ppm to FeCrNi-based austenitic stainless steels is recognized to be beneficial to the creep resistance, hot workability, and intergranular corrosion resistance [13,14,15,16,17,18]. However, the solubility of B in the austenitic stainless steels is very limited—as low as approximately 100–150 ppm depending on the matrix composition [9,13,19]; thus, the addition of an excessive amount of B inevitably forms M_2_B (M stands for metal; Cr and/or Fe) type phase, which is known to degrade the mechanical and corrosion properties [4,5,6,7,8,13,20]. The corrosion damage of the stainless steel racks can accelerate the fracture of the structure; thus, the corrosion behavior of BSSs has been investigated and well documented. Loria et al. [13] reported the general corrosion behavior and intergranular corrosion susceptibility of type 304 stainless steel with 0.5 wt % B and without B in sulfuric acid solution. He et al. [8] investigated the general and localized corrosion behavior of BSSs (UNS S30464 and UNS S30465) in simulated groundwater at 60–90 °C. Lister et al. [7] also examined the general and pitting corrosion resistance of UNS S30464-S30466 alloys (made through powder metallurgy) and type 316 stainless steel with 1 wt % B (made through ingot metallurgy) in aqueous solutions containing Cl^−^ and NO_3_^−^. In addition, Moreno et al. [6] focused on the pitting corrosion resistance of UNS S30466 alloy in aqueous solutions with NaCl and NaCl + Na_2_S. Upadhyay et al. [20] investigated the localized corrosion resistance of type 304 stainless steels with (1.2 wt %) and without B through electrochemical noise analyses and polarization tests. A consensus was found that lower corrosion resistance was obtained in the alloys with higher B content.

Although several researchers have investigated the various types of corrosion of BSSs, little work has been done on the passive behavior of BSSs. In general, the BSSs applied for wet spent fuel storage racks are installed in an on-site spent fuel storage pool, and they are exposed to a relatively mild corrosive environment with low Cl^−^ concentration and near-neutral pH. Under this condition, the overall corrosion resistance of the BSS rack primarily depends on the protectiveness of the passive film; thus, it is necessary to investigate the passive behavior of BSSs. Therefore, the present paper aims to investigate the passive behavior and the resistance to passivity breakdown of type 304 stainless steels (Fe_balance_18Cr12Ni1.5Mn-based alloys, in wt %) containing 0.19–1.76 wt % B, which were manufactured through conventional ingot metallurgy.

## 2. Materials and Methods

### 2.1. Materials

The investigated alloys were Fe_balance_18Cr12Ni1.5Mn-based alloys (type 304 stainless steels) containing 0.19–1.76 wt % B. The detailed chemical compositions of the alloys are given in Table 1. The alloys were fabricated through conventional ingot metallurgy. The ingots (50 kg) were reheated at 1150 °C for 1 h and then hot-rolled into plates with a thickness of 40 mm. The hot-rolled plates were solutionized at 1050 °C for 1 h followed by water quenching. The temperatures for reheating and solutionization were determined based on thermodynamic calculations, which were conducted using ThermoCalc software with the TCFE 7.0 database [21]. Based on the compositions in Table 1, the equilibrium between liquid, FCC, BCC, Cr_2_B, and Cr_23_C_6_ phases was estimated.

For microstructure observation, the alloy specimens (15 mm × 10 mm × 3 mm) were polished using a diamond suspension with a particle size of 1 μm and then etched using a mixed solution of 30 mL HCl + 20 mL HNO_3_ + 50 mL ethanol. The microstructures of the specimens were observed using an optical microscope (Epiphot, Nikon, Japan) and a scanning electron microscope (SEM, JSM-7100F, JEOL, Tokyo, Japan). In addition, the chemical composition of the second phase was investigated using electron probe microanalysis with wavelength dispersive spectrometry (EPMA-WDS, JXA-8530F, JEOL). Based on the micrographs, fractions and sizes of the second phases were measured using Image-Pro Plus 7.1 software (Media Cybernetics, Silver Spring, MD, USA). For the analyses of the second phase formed after solution treatment, a transmission electron microscope (TEM, JEM-2100F, JEOL, Japan) operating at 200 kV was used. In order to identify the second phase directly, a dual beam system (NOVA 200, FEI Company, Hillsboro, OR, USA) composed of both focused ion beam (FIB) and high-resolution SEM columns was used. Cross-section milling was performed with 30 kV Ga ions. Pt was deposited on the interface between the Omni-probe and sample, and the final cuts were made by FIB. The standard lift-out technique was used where the specimen was removed from the trench using an Omni-probe and placed on a Cu grid for TEM observation.

### 2.2. Pitting Corrosion Resistance

The resistance to pitting corrosion of the alloys was evaluated through polarization and immersion tests in aqueous solutions containing Cl^−^. The polarization tests were conducted in 10 ppm and 58 ppm NaCl solutions at 20 ± 1 °C, and the potential sweep rate was 2 mV s^−1^. After the test, the pit initiation sites were observed using SEM and a surface profiler (Wyko NT8000, Veeco, Plainview, NY, USA [22]). The immersion tests were conducted in 3.5 wt % (=0.6 M) NaCl solution for 35 days at 20 ± 1 °C. For the immersion tests, the specimens (40 mm × 10 mm × 3 mm) were polished using a 1 μm sized diamond suspension.

### 2.3. Passive Film Properties

Passive behavior and passive film properties were investigated. For these investigations, a borate–phosphate–citric buffer solution with pH 8.5 (0.2 M boric acid + 0.05 M citric acid + 0.1 M tertiary sodium phosphate) was used, which is proven to form a stable and thick passive film on Fe-based alloys [23]. The polarization behavior of the alloy was examined through potentiodynamic polarization tests in the buffer solution at 20 ± 1 °C at a potential sweep rate of 2 mV s^−1^ in order to measure the potential range for the stable passive state and passive current density (i_passive_).

Then the chemical and electronic properties of the passive films were investigated. The chemical composition of the passive film was examined through X-ray photoelectron spectroscopy (XPS, PHI 5000 VersaProbe, ULVAC-PHI, Kanagawa, Japan) using an Al Kα anode X-ray source (150 W, 15 kV, hν = 1486.6 eV). For the XPS analysis, the passive films were formed on the BSSs in the buffer solution (pH 8.5) by applying constant anodic potential of 0 V_SCE_ for 3 h.

Regarding the electronic properties of the passive film, the point defect density of the space charge layer of the passive film was investigated through Mott–Schottky analysis. For this, the passive film was potentiostatically grown by applying constant anodic potential of 0.6 V_SCE_ for 1 h in the borate–phosphate–citric buffer solution (pH 8.5), and then the capacitance of the passivated layer was measured at a frequency of 1000 Hz with an imposing sinusoidal voltage perturbation of ±0.01 V (peak-to-peak) during the negative potential sweep from 0.6 to −0.7 V_SCE_ with a potential sweep rate of 0.01 V step^−1^.

All of the polarization tests and capacitance measurements (Mott–Schottky analysis) were conducted in a standard three-electrode setup with the metal specimen being a working electrode, a Pt plate (50 mm × 120 mm × 0.1 mm) as a counter electrode, and a saturated calomel reference electrode (SCE) as a reference electrode; the electrochemical tests were controlled by a potentiostat (Reference 600, GAMRY Instruments, Philadelphia, PA, USA). For the working electrode, the specimens (10 mm × 10 mm × 3 mm) were mounted in cold epoxy resin and then mechanically ground using SiC emery paper up to 2000 grit. The polarization test was performed on an exposed area of 0.2 cm^2^, which was controlled using electroplating tape. The polarization tests were performed on each specimen 3–5 times, and the capacitance of the passive layer was repetitively measured on each specimen three times in order to confirm reproducibility.

## 3. Results and Discussion

### 3.1. Microstructure

**Figure 1:**
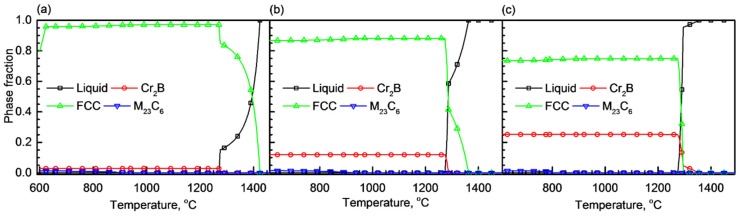
Equilibrium phase diagrams of (**a**) B019, (**b**) B078, and (**c**) B176 alloys calculated using Thermo-Calc software.

**Figure 2:**
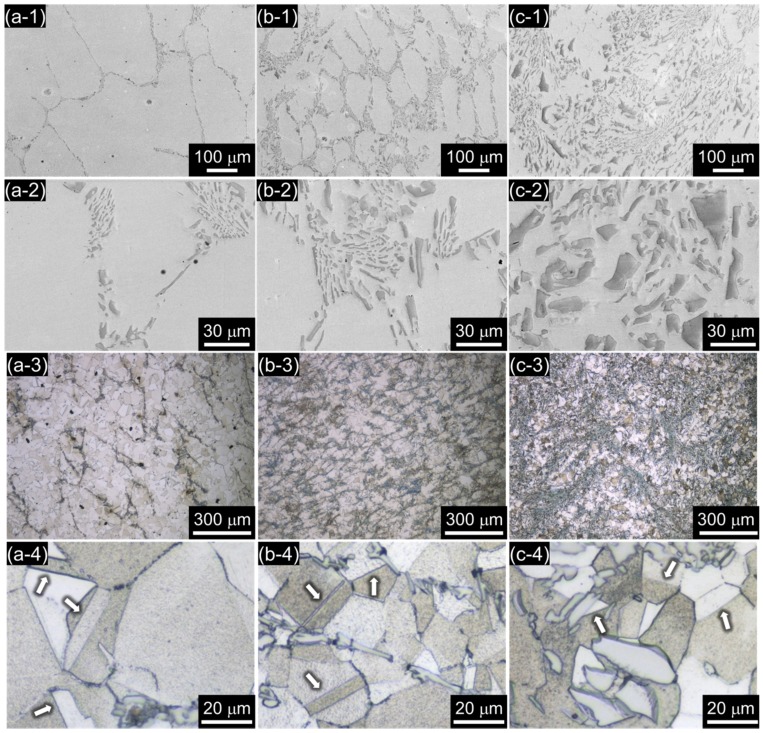
Microstructures of (**a**) B019, (**b**) B078, and (**c**) B176 alloys. SEM images of the alloys taken at (**a-1**,**b-1**,**c-1**) low and (**a-2**,**b-2**,**c-2**) high magnification, and optical micrographs of the alloys taken at (**a-3**,**b-3**,**c-3**) low and (**a-4**,**b-4**,**c-4**) high magnification.

Figure 1 shows the equilibrium phase fractions as a function of temperature. In the case of B019 alloy, the pure liquid state is stable above 1424 °C, and the fraction of austenite phase increases as the temperature decreases. Therefore, during the cooling process from the liquid, austenite phase is firstly formed and then Cr and B become enriched in the liquid phase. When the remaining liquid reaches a critical temperature, a eutectic reaction occurs in which austenite and Cr_2_B are simultaneously produced in the liquid. These eutectic reactions similarly occur in B078 and B176 alloys. The melting points of the B019, B078, and B176 alloys are 1270, 1273, and 1274 °C, respectively. The differences in the melting points are induced by other alloying elements distributed in the austenite phase. In the case of B176 containing 1.76 wt % B, the eutectic reaction mainly occurred without producing a pro-eutectic phase. The results of the reactions were confirmed in the microstructure observations in Figure 2.

**Figure 3:**
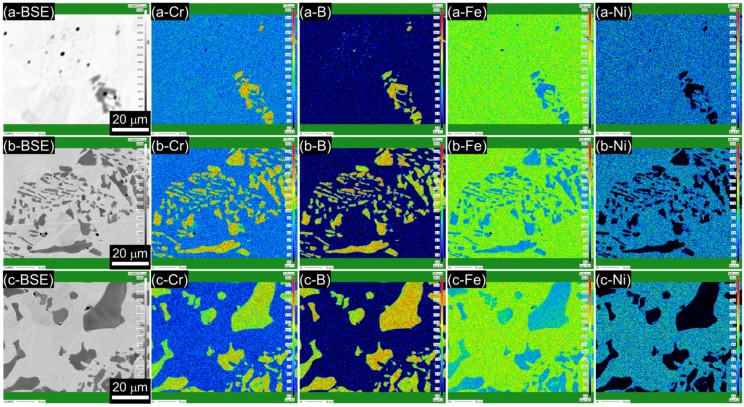
Elemental X-ray mapping of Cr, B, Fe, and Ni along with the corresponding back-scattered electron (BSE) images of the (**a**) B019, (**b**) B078, and (**c**) B176 alloys.

**Figure 4:**
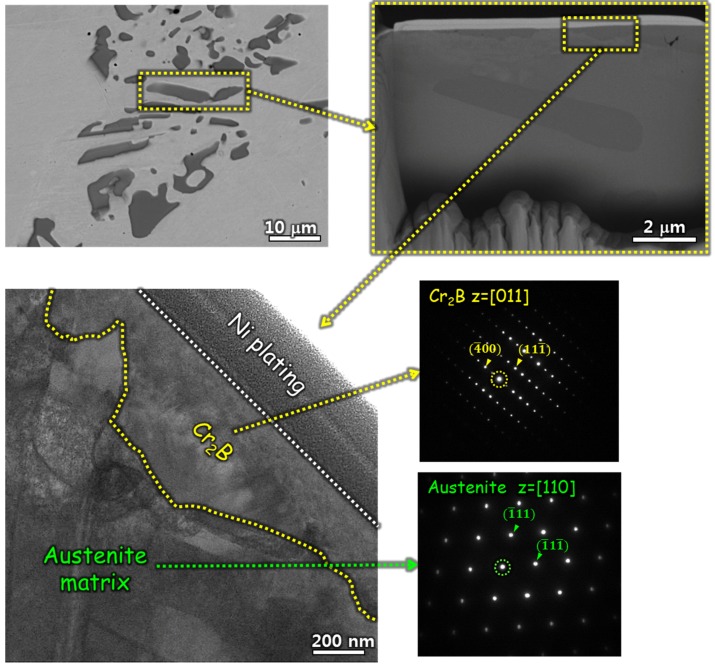
Procedure of focused ion beam (FIB) sampling, bright field (BF) imaging, and corresponding selected area diffraction (SAD) patterns of Cr_2_B and the austenite matrix taken from the B078 alloy.

Figure 2a-1–c-2 is SEM images of B019, B078, and B176 alloys showing the distribution of the second phase in the matrix. The second phases were irregular in shape as well as size (ranging over 2–30 μm) and were not homogeneously distributed over the matrix. The microstructure of the B019 alloy (Figure 2a-1–a-4) shows the mixture of the second phases and austenite formed by the eutectic reaction around the austenite that first formed during cooling. On the other hand, in the case of the B176 alloy (Figure 2c-1–c-4), the second phase was distributed rather homogeneously in the austenite matrix. The measured fractions of the second phases in the B019, B078, and B176 alloys were 1.68 ± 0.49, 9.73 ± 0.28, and 22.66 ± 0.8 vol %, respectively, which are similar to the predicted values from the thermodynamic calculation.

In the B019 alloy (Figure 2a-2), second phases with needle-like shapes were observed, and as the B content increased to 0.78 and 1.76 wt % (Figure 2b-2 and c-2, respectively), both the volume fraction and number of the second phases increased, and the platelike-shaped second phases were frequently found in the B176 alloy.

The equilibrium phase diagrams in Figure 1 suggest that the relatively dark phase shown in the SEM images (Figure 2) is Cr_2_B and that the matrix is austenite, which was confirmed by the composition analysis through the EPMA (Figure 3). Figure 3a–c shows back-scattered electron (BSE) images and the elemental maps (Cr, B, Fe, and Ni) of the B019, B078, and B176 alloys. In the BSE images (Figure 3a-BSE–c-BSE), the second phase appears in dark gray in comparison with the matrix (light gray), which indicates that the second phase contains heavier elements than the matrix. Figure 3a-Cr–c-Fe clearly shows that Cr and B are enriched with the second phase where Fe is slightly depleted. Ni is rarely detected in the second phase as shown in Figure 3a-Ni–c-Ni.

Shown in Figure 4 are SEM micrographs of FIB sampling, bright field (BF) TEM images, and selected area diffraction patterns (SADP) of the second phase as well as the matrix taken from the B078 specimen. Based on the analyses of SADP, the second phase was confirmed to be Cr_2_B with orthorhombic structure (space group: F*ddd*) and the lattice parameters of Cr_2_B are *a* = 0.4275 nm, *b* = 0.7452 nm, and *c* = 1.4795 nm.

Because the second phase is the (Cr,B)-enriched phase, the concentrations of Cr and B in solid solution state in the matrix consequently decrease as the volume fraction of Cr_2_B increases. Most of the alloyed B was consumed by forming Cr_2_B; thus, the concentrations of B in solid solution state of the austenite matrices of the three BSSs were calculated to be less than approximately 2.0 × 10^−5^ wt %. Accordingly, the Cr content in the matrix decreased as the alloyed B content increased. The Cr contents of the austenitic matrices of B019, B078, and B176 alloys at solution treatment temperature were calculated to be 16.67, 13.65, and 9.21 wt %, respectively. On the other hand, Ni and Mn, which did not participate in forming Cr_2_B, were anticipated to be enriched in the austenitic matrix. Indeed, the calculated Ni contents of the austenitic matrices increased from 12.34 wt % for the B019 alloy to 14.85 wt % for the B176 alloy. Those of Mn also increased from 1.38 wt % for the B019 alloy to 1.68 wt % for the B176 alloy.

Figure 2a-3–c-4 is optical micrographs of the three alloys, which were polished and etched in acidic solution to identify the grain boundary. In the three alloys, the annealing twins (marked by arrows in Figure 2a-4–c-4) were frequently observed in the polycrystallized matrix, confirming that the matrix was recrystallized austenite. It is noted that the average grain diameter decreases as the B content increases, because the eutectic Cr_2_B effectively blocks grain boundary migration during the heat treatment.

### 3.2. Pitting Corrosion Resistance

**Figure 5:**
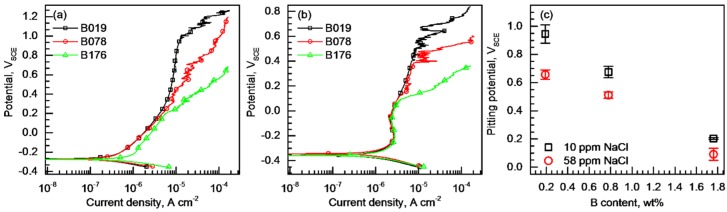
Potentiodynamic polarization curves of the alloys measured in (**a**) 10 ppm and (**b**) 58 ppm NaCl solutions at 20 ± 1 °C at a potential sweep rate of 2 mV s^−1^; (**c**) Variation of the average pitting potentials of the alloys as a function of the B content.

Figure 5a,b exhibits the potentiodynamic polarization curves of the B019, B078, and B176 alloys measured in 10 ppm and 58 ppm NaCl solutions, respectively. As shown in Figure 5a,b, the three BSSs passivate in the dilute NaCl solutions under the open circuit condition and do not exhibit active–passive transition. In the 10 ppm NaCl solution (Figure 5a), the E_corr_ values of the three alloys were −0.274 V_SCE_, and those were slightly shifted to the lower potential of −0.353 V_SCE_ in the NaCl solution with increased NaCl concentration (Figure 5b). For the three alloys, stable passivity appeared only in the limited potential range from the E_corr_ to the pitting potential (E_pit_). The average E_pit_ was calculated from repetitive polarization tests and is plotted in Figure 5c as a function of the B content. Higher E_pit_ was obtained in more dilute NaCl solution, as expected, and it was clear that the E_pit_ was linearly lowered with an increase in the B content in the alloys. Therefore, it could be concluded that the resistance to pitting corrosion of the BSSs was degraded as the B content increased.

**Figure 6:**
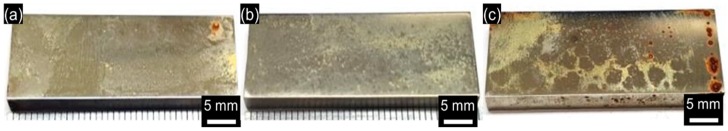
Corrosion morphologies of (**a**) B019, (**b**) B078, and (**c**) B176 alloys after immersion tests in a 0.6 M NaCl solution at 20 ± 1 °C for 35 days.

Figure 6 shows the corroded surfaces of the three alloy specimens after immersion in a 3.5 wt % NaCl solution for 35 days. Figure 6 confirms the decrease in the resistance to pitting corrosion along with the increase in the B content in the alloys. The photos demonstrate that pitting corrosion is the primary corrosion type in this Cl^−^-containing environment, and the number of pits and damaged area increase as the B content increases in the alloys.

**Figure 7:**
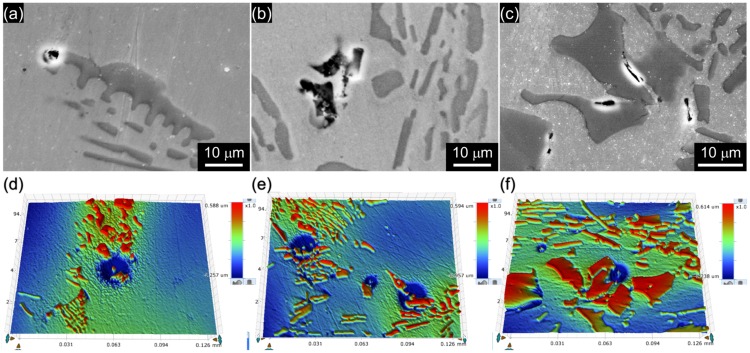
SEM images of the pit initiation sites of (**a**) B019, (**b**) B078, and (**c**) B176 alloys. Three-dimensional surface topographies of the pit initiation sites of (**d**) B019, (**e**) B078, and (**f**) B176 alloys.

Figure 7a–c shows SEM images of the pit initiation sites observed in B019, B078, and B176 specimens, respectively. In all of the alloys, pits are initiated at the matrix adjacent to the Cr_2_B and propagated into the matrix. Figure 7d–f exhibits 3-dimensional surface topographies of the pitted BSS specimens measured through a surface profiler. In the topographies, the higher phase (red color) than the matrix (green and blue color) is Cr_2_B due to its higher hardness than that of the matrix [24]. Figure 7d–f also clearly demonstrate that the pitting corrosion occurs at the boundary between the Cr_2_B and the matrix, which corrodes while the Cr_2_B remains intact [7,11,20].

### 3.3. Passive Film Analysis

**Figure 8:**
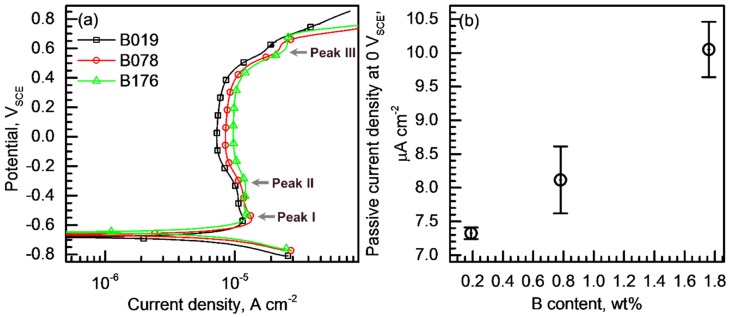
(**a**) Potentiodynamic polarization curves of the alloys measured in a borate–phosphate–citric buffer solution with pH 8.5 (0.2 M boric acid + 0.05 M citric acid + 0.1 M tertiary sodium phosphate) at 20 ± 1 °C at a potential sweep rate of 2 mV s^−1^; (**b**) Variation of the average passive current density values of the alloys as a function of the B content.

The passive behavior of the BSSs was evaluated. Figure 8a shows potentiodynamic polarization curves of the alloys measured in the borate–phosphate–citric buffer solution at pH 8.5. All of the alloys exhibit passive behavior in this solution without active–passive transition, and the E_corr_ values of the alloys are approximately −0.66 V_SCE_. In the polarization curves of the BSSs, there are three current peaks at −0.55, −0.33, and 0.57 V_SCE_, indicated by arrows in Figure 8a. Peak I at −0.55 V_SCE_ is attributed to the oxidation of Fe to Fe^2+^, and Peak II at −0.33 V_SCE_ is due to the reoxidation of Fe^2+^ to Fe^3+^. Peak III at 0.57 V_SCE_ reflects the reoxidation reaction of Cr^3+^ to Cr^6+^. In addition, the rapid increase in the current density above approximately 0.65 V_SCE_ is due to oxygen evolution (that is, transpassive reaction) [25,26,27,28]. The polarization curves exhibit that the potential range for the stable passivity extends from E_corr_ (approximately −0.66 V_SCE_) to approximately 0.65 V_SCE_. In the passive potential range, the lowest i_passive_ is observed at approximately 0 V_SCE_. The average i_passive_ values (measured at 0 V_SCE_) of the alloys were calculated from the repetitively measured polarization curves 3–5 times and plotted versus the B content (Figure 8b). The minimum i_passive_ value increased from 7.32 to 10.05 μA cm^−2^ as the B content increased from 0.19 to 1.76 wt %; thus, it was concluded that the passive film with the highest resistance was formed on the BSS containing the lowest B content. It is worth mentioning the decrease in the grain size of the austenite matrix of the BSSs as shown in Figure 2a-3–c-4. The grain refinement is known to accelerate passivation, resulting in the formation of a thick and dense passive film [29,30,31]; thus, the i_passive_ is generally lowered when the grain size of the matrix decreases [32]. In this case, however, the i_passive_ of the BSSs apparently increased although the grain size of the austenite matrix decreased; thus, it is reasonable to conclude that the change in the grain size is not the dominant factor for determining the magnitude of the i_passive_.

**Figure 9:**
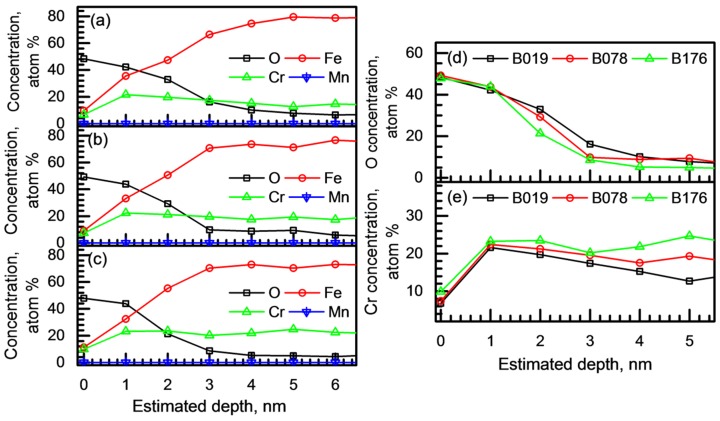
Chemical composition depth profiles of the passive films of (**a**) B019, (**b**) B078, and (**c**) B176 alloys analyzed through XPS; (**d**) O and (**e**) Cr concentration profiles of the passive films formed on the alloys. The passive films were formed in a borate–phosphate–citric buffer solution (pH 8.5) by applying constant anodic potential of 0 V_SCE_ for 3 h.

The chemical composition and structure of the passive film were examined using XPS. For this analysis, the passive film was potentiostatically formed in the borate–phosphate–citric buffer solution (pH 8.5), and the film formation potential was determined to be 0 V_SCE_, at which the lowest i_passive_ was observed as presented in Figure 8a. Figure 9a–c shows the concentration depth profiles of the B019, B078, and B176 alloys, respectively. Figure 9 demonstrates that stable passive films with similar structure and chemical composition were formed on the three BSSs. The passive films were primarily composed of Fe, Cr, and O with a small amount of Mn (less than 0.05 atom %). A notable difference among the passive films of the three BSSs was observed in the film thickness. The thickness of the passive film can be estimated from the concentration depth profile of O (Figure 9d) by taking the depth from the surface at which the 50% value of the O amplitude appears [31,33,34]. The thickness of the passive film formed on the B176 alloy was calculated to be 1.823 nm, while that on the B019 alloy was 2.469 nm. That is, a thinner passive film was formed on the BSS with higher B content. In addition, the Cr content was also affected by the B content in the matrix. As shown in Figure 9e, the Cr contents at the film surface of the B019, B078, and B176 alloys were 6.80, 7.39, and 9.89 atom %, respectively, and the Cr content inside the passive film was also slightly higher in the B176 alloy than in the other alloys.

Then, the point defect density in the passive film was measured through Mott–Schottky analysis. Generally, the passive film of stainless steel formed in an aqueous solution is known to contain large numbers of point defects such as oxygen vacancies (V_O_^2+^), metal vacancies (V_M_^x−^), and cation interstitials (M_i_^x+^); thus, the passive film behaves as an extrinsic semiconductor. The point defect density of the space charge layer in the passive film can be calculated through the capacitance measurement, which is Mott–Schottky analysis. For Mott–Schottky analyses, the specific interfacial capacitance (*C_total_*) of the passivated surface is obtained using *C_total_ =* 1/*ωZ″*, where *ω* is the angular frequency and *Z″* is the imaginary part of the specific impedance. The measured capacitance (*C_total_*) is a series combination of the double layer capacitance (Helmholtz layer capacitance, *C_H_)* and space charge layer capacitance (*C_SC_*). The *C_SC_* of the n-type semiconductor and the relationship between *C_total_*, *C_H_*, and *C_SC_* are given as follows:

(1) $$\frac{1}{C_{SC}^{2}}=\frac{1}{C_{total}^{2}}-\frac{1}{C_{H}^{2}}=\left(\frac{2}{\varepsilon \varepsilon_{0}eN_{D}}\right)\left(E_{app}-E_{fb}-\frac{k_{B}t}{e}\right)$$

where *ε* is the dielectric constant of the passive film (15.6 for the passive film of stainless steel [35,36]), *ε*_0_ is the vacuum permittivity (8.854 × 10^14^ F cm^−1^), *e* is the electron charge, *E_app_* is the applied potential, and *k* is the Boltzmann constant. Thus, for an n-type semiconductor, a graph of *C_SC_*^−2^ versus *E_app_* should be linear with a positive slope and *C_SC_*^−2^ is inversely proportional to donor density (*N_D_*). In the Mott–Schottky relationship, *C_H_* is sufficiently higher than *C_SC_*; therefore, it can be neglected in a series of combinations with the *C_SC_*. Thus, the measured capacitance (*C_total_*) can be assumed to be equal to *C_SC_*.

**Figure 10:**
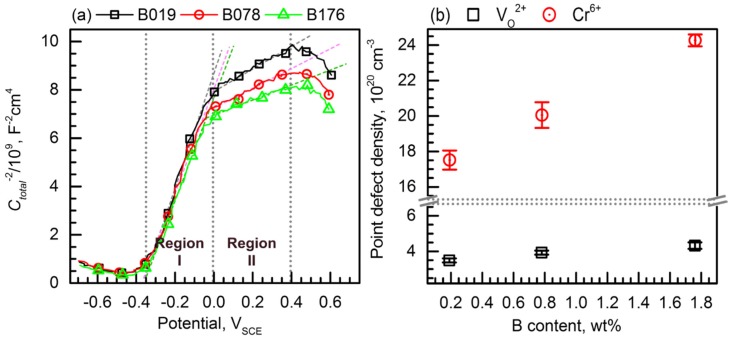
(**a**) Mott–Schottky plots for the passive films formed on the borated stainless steels (BSSs). The passive films were formed in a borate–phosphate–citric buffer solution (pH 8.5) by applying constant anodic potential of 0.6 V_SCE_ for 1 h, and the capacitance of the passivated layer was measured at a frequency of 1000 Hz with an imposing sinusoidal voltage perturbation of ±0.01 V (peak-to-peak); (**b**) Variations of the densities of the point defects (V_O_^2+^ and Cr^6+^) as a function of the B content.

Mott–Schottky plots of the BSSs are presented in Figure 10a, as measured in the borate–phosphate–citric buffer solution at pH 8.5. For the Mott–Schottky analysis, the passive film was formed by applying constant anodic potential of 0.6 V_SCE_ for 1 h and capacitance was then measured at a constant frequency of 1 kHz with an imposing sinusoidal voltage perturbation of ±10 mV in a potential range from 0.6 V_SCE_ to −0.7 V_SCE_. The XPS analysis (Figure 9) confirmed that the passive films formed on the BSSs were (Fe,Cr)-oxide, which is known to have n-type semiconductivity. In accordance with the Mott–Schottky relation (Equation (1)), the n-type semiconductor passive film exhibits a positive slope (*∆C_total_^−2^*/*∆V*) in the Mott–Schottky plot, and the dominant and detective point defects in the n-type semiconductor passive film are *V_O_*^2*+*^ (shallow donor) and Cr^6+^ (deep donor). In order to investigate the densities of both donors, film formation for Mott–Schottky analysis was conducted by applying anodic potential between peak III (Cr^3+^ → Cr^6+^, shown in Figure 8a) and the transpassive potential where oxygen evolution occurred.

The Mott–Schottky plots of the BSSs shown in Figure 10a exhibit two potential sections showing linear increase, between −0.35 and 0 V_SCE_ (Region I) and between 0 and 0.4 V_SCE_ (Region II). Using the positive *∆C_total_^−2^*/*∆V* values in Regions I and II, the shallow and deep donor densities can be estimated, respectively.

The average point defect density values of the BSSs are presented in Figure 10b as a function of the B content. Figure 10b clearly shows that the densities of both shallow and deep donors increase with the increase in the B content. The average shallow donor density of the B019 alloy was 3.49 × 10^20^ cm^−3^ and that of the B176 alloy was 4.32 × 10^20^ cm^−3^. In addition, the average deep donor density increased from 17.51 × 10^20^ cm^−3^ for the B019 alloy to 24.27 × 10^20^ cm^−3^ for the B176 alloy. The high point defect density in the passive film implies a large amount of charge carrier in the passive film which well explains the higher i_passive_ of the B176 alloy than that of the B019 alloy in the polarization curves, as shown in Figure 8b. In addition, Figure 10b also demonstrates that the alloy containing more B forms more defective and, hence, less protective passive film; thus, the results from Figure 10b are partly responsible for the degraded resistance to pitting corrosion of the B176 alloy shown in Figure 5 and Figure 6.

The noticeable point in Figure 10b is the change in the Cr^6+^ concentration. The density of Cr^6+^ in the passive film significantly increased with the increase in B content in the BSSs, which corresponded to the XPS analysis result (Figure 9e). It is generally accepted that the passive film of stainless steel (FeCr-based alloys) containing higher Cr is more protective [28,36,37], resulting in the enhancement of the resistance against passive breakdown. However, in the case of BSSs, the passive film formed on the B176 alloy exhibited the lowest resistance to pitting corrosion although the film contained the highest Cr concentration. This discrepancy can be explained as follows: First, the overall resistance against pitting corrosion of the BSSs shown in Figure 5, Figure 6 and Figure 7 is primarily determined by the surface heterogeneity (i.e., Cr_2_B), which provides the pit initiation site, in comparison with the protective ability of the passive film. Second, the detrimental contribution of Cr_2_B to the overall passivation is considered. As discussed in Section 3.1, the Cr content in the solid solution state in the austenite matrix decreased from 16.67 to 9.21 wt % as the B addition increased from 0.19 to 1.76 wt % because of the Cr_2_B formation. Thus, the higher Cr concentration in the passive film on the BSS with higher B content is considered as evidence of the passivation of Cr_2_B. However, the electrical conductivity of the Cr_2_B is lower than that of the austenite matrix [38], and the volume fraction of Cr_2_B remarkably increases as the B addition increases; thus, the formation of a stable and continuous passive film on the B-containing alloy is inhibited. As a result, a thin (Figure 9d) and defective passive film is formed on the alloy with high B content.

## 4. Conclusions

The resistance to pitting corrosion and passive behavior of type 304 stainless steels (Fe_balance_18Cr12Ni1.5Mn-based alloys, in wt %) containing 0.19–1.76 wt % B, which were manufactured through conventional ingot metallurgy, were investigated. The pitting corrosion resistance was evaluated through potentiodynamic polarization and immersion tests in aqueous solutions with Cl^−^. The passive behavior was examined in a borate–phosphate–citric buffer solution at pH 8.5 through potentiodynamic polarization tests. The physicochemical and electronic properties of the passive film were examined using XPS and Mott–Schottky analyses. On the basis of the tests, the following conclusions could be drawn.

1. The borated stainless steels were composed of austenitic matrix and Cr_2_B phase. As the B content increased from 0.19 to 1.76 wt %, the volume fraction of Cr_2_B increased from 1.68 to 22.66 vol %, and the concentration of Cr in solid solution state in the austenitic matrix was lowered from 16.67 to 9.21 wt %. In addition, the grain size of the austenite matrix decreased as the Cr_2_B fraction increased.
2. In various NaCl solutions, lower pitting corrosion resistance was observed in the alloy with higher B content. The pits were initiated at the matrix adjacent to the Cr_2_B and propagated into the matrix.
3. Regarding the passive behavior, the passive current density increased as the B content in the alloy increased. The passive films of the borated stainless steels formed in borate–phosphate–citric buffer solution (pH 8.5) were (Fe,Cr)-oxides. With an increase in the B addition, the passive film thickness decreased from 2.5 to 1.8 nm, and the Cr content in the passive film slightly increased. Furthermore, Mott–Schottky analysis confirmed that more defective passive film was formed on the alloy with higher B content.
4. The reduced resistance to pitting corrosion of the B-bearing type 304 stainless steel along with the increase in the B content was due to the formation of a more defective and thinner passive film and a larger number of pit initiation sites in the matrix.

## Figures and Tables

**Table 1:** Chemical compositions (in wt %) of the investigated alloys.

Alloy	Fe	Cr	Ni	Mn	Al	C	Si	B
B019	Balance	17.7	11.9	1.35	0.024	0.056	0.25	0.19
B078		18.4	12.3	1.55	0.035	0.066	0.26	0.78
B176		18.3	12.4	1.57	0.039	0.073	0.28	1.76
